# Impact of respiratory disease, diarrhea, otitis and arthritis on mortality and carcass traits in white veal calves

**DOI:** 10.1186/1746-6148-9-79

**Published:** 2013-04-15

**Authors:** Bart Pardon, Miel Hostens, Luc Duchateau, Jeroen Dewulf, Koen De Bleecker, Piet Deprez

**Affiliations:** 1 Department of Large Animal Internal Medicine, Faculty of Veterinary Medicine, Ghent University, Salisburylaan 133, Merelbeke, 9820, Belgium; 2 Veterinary Epidemiology Unit, Department of Reproduction, Obstetrics and Herd Health, Faculty of Veterinary Medicine, Ghent University, Salisburylaan 133, Merelbeke, 9820, Belgium; 3 Department of Physiology and Biometry, Faculty of Veterinary Medicine, Ghent University, Salisburylaan 133, Merelbeke, 9820, Belgium; 4 Animal Health Service-Flanders, Industrielaan 29, Torhout, 8820, Belgium

**Keywords:** Bovine respiratory disease, Carcass weight, Diarrhea, Economics, Mortality, Veal calves

## Abstract

**Background:**

Little is known on the effects of common calf diseases on mortality and carcass traits in the white veal industry (special-fed veal), a highly integrated production system, currently criticized for the intensive pro- and metaphylactic use of antimicrobials. The objective of the present study was to determine the impact of bovine respiratory disease (BRD), diarrhea, arthritis and otitis on the economically important parameters of mortality, hot carcass weight (HCW), carcass quality, fat cover and meat color. For this purpose, a prospective study on 3519 white veal calves, housed in 10 commercial herds, was conducted. Case definitions were based on clinical observation by the producers and written treatment records were used.

**Results:**

Calves received oral antimicrobial group treatments in the milk during 25.2% of the production time on average. With an increasing percentage of the production cycle spent on oral antimicrobials, HCW reduced, whereas the odds for insufficient fat cover or an undesirable red meat color both decreased. Of the calves, 14.8%, 5.3%, 1.5% and 1.6% were individually diagnosed and treated for BRD, diarrhea, arthritis and otitis, respectively. Overall, 5.7% of the calves died and the mortality risk was higher in the first weeks after arrival. Calves that experienced one BRD episode showed a 8.2 kg reduction in HCW, a lower fat cover and an increased mortality risk (hazard ratio (HR) = 5.5), compared to calves which were not individually diagnosed and treated for BRD. With an increasing number of BRD episodes, these losses increased dramatically. Additionally, calves, which experienced multiple BRD episodes, were more likely to have poor carcass quality and an undesirable red meat color at slaughter. Arthritis increased the mortality risk (HR = 3.9), and reduced HCW only when associated with BRD. Otitis did only increase the mortality risk (HR = 7.0). Diarrhea severely increased the mortality risk (HR = 11.0), reduced HCW by 9.2 kg on average and decreased carcass quality.

**Conclusions:**

Despite the massive use of group and individual treatments to alleviate the most prevalent health issues at the fattening period, the effects of BRD, diarrhea, otitis and arthritis on survival and performance are still considerable, especially in cases of chronic pneumonia with or without arthritis. Controlling calf health by effective preventive and therapeutic strategies and in particular the prevention of chronic BRD is key for the profitability of veal operations.

## Background

The white veal industry (special-fed veal) is specialized in raising, predominantly male, calves of different breeds on a low iron milk replacer diet to obtain pale veal meat [1]. European veal production stood at 5.8 million calves in 2008, with the main producers being France, The Netherlands and Italy [1]. Also, in the United States special-fed veal represents a 1 billion dollar industry [2]. Several studies in feedlots and dairy operations have shown that calf diseases have an important impact on economic parameters such as mortality, weight gain and carcass traits and that this impact differs according to management and treatment strategies [3-13]. Despite the large production scale and high degree of integration in the veal industry, little is known on the effects of common diseases on these economic parameters in contemporary, group housed, white veal calves [14-16]. The most frequent diseases in white veal operations are bovine respiratory disease (BRD), diarrhea, arthritis and otitis [17]. Whereas most previous studies in different cattle production systems focused on BRD, there is little information on the impact of diarrhea, arthritis and otitis in veal or beef production. Available studies on veal calves either addressed individual housing systems, which are nowadays prohibited in the European Union, or only determined short term disease effects related to the clinical period [14,15,18-21]. In contrast, more recent studies in other cattle production systems, have shown that several of these short term disease effects are less pronounced or no longer meaningful at slaughter [4,11,22,23]. Contemporary veal management relies on the intensive use of oral pro- and metaphylactic group antimicrobial treatments, which is highly criticized by the European authorities at present [24]. A prophylactic treatment is defined as the treatment of healthy animals to prevent disease from occurring, whereas a metaphylactic treatment implies the simultaneous treatment in a shared compartment of clinically healthy animals and animals that showed clinical symptoms of the disease [25]. To what extent calves, that still develop disease under such management, have poorer production results compared to their pen mates is unknown.

Therefore, the objective of the present study was to determine the impact of BRD, diarrhea, arthritis and otitis on mortality and carcass traits (hot carcass weight (HCW), carcass quality, carcass color and fat cover) in white veal calves, raised under contemporary management.

## Methods

### Herds and animals

A prospective cohort study was set up to determine the impact of veal calf diseases on mortality and carcass traits. The study group consisted of 3519 white veal calves, housed in 10 commercial veal farms in Northern Belgium. Participating herds were conveniently selected based upon willingness to cooperate, but independent of any disease history. In the veal industry the diet differs in between breeds and therefore calves of the same breed or confirmation (e.g. crossbreds) are grouped within a farm. Therefore, a veal production type stands for the combination of the breed effect and the specific diet that breed receives. In the present study the sample was stratified on production type (3 dairy, 4 beef and 3 crossbred herds). Dairy calves belonged to the black or red Holstein Friesian (BHF and RHF) breed, beef calves were mostly Belgian Blue (BB) and crossbreds mainly involved HFxBB. Selected herds belonged to six different integrations, including the three largest integrations in Belgium. An integration is a company that combines all steps of the production chain by having its own feed plant and slaughterhouse and by placing its calves in veal herds owned by producers that fatten these calves for the integration on contract. One all in/all out production cycle per herd (= 1 cohort) was monitored from calf arrival to slaughter, and all calves from that cohort were included in the study. Herds gradually entered the study between January 2008 and October 2009. Calves originated from multiple herds and were transported within 24 h from the herd of origin to the veal herds, at the minimum age of 14 days old, after a short stay at a sorting center. Calves were individually housed during the first 6 weeks and thereafter group housed in galvanized pens on slatted floors in compliance with European legislation (Council Directives 91/629/EEC and 97/2/EC). Calves received an all-liquid milk diet, supplemented with solid feed (fibers and concentrates according to European legislation). The milk diet was different between the three production systems: beef calves received predominantly a high quality skimmed milk powder, whereas the skimmed milk diet of dairy and crossbred calves was progressively changed to a lower quality milk powder, based on whey and vegetable proteins, in 8 weeks’ time. Calves were not vaccinated against any pathogen.

### Data collection

Calves were individually identified by ear tag, according to Belgian law. Calf entry characteristics (birth date, arrival date, breed, gender) were collected from the Belgian cattle registration system (SANITEL). The date of mortality and the cause as determined by necropsy were recorded. The used definitions for each cause of death were as published previously [17]. All individual (calf identity, indication and drug) and group treatments, administered by producer or veterinarian, were recorded daily on written treatment records. Clinical signs on which the producers based their decision to individually treat an animal were the presence of liquid feces for diarrhea, swollen joints and/or lameness for arthritis and head tilt for otitis. For respiratory disease producers used mental state, appetite, nasal discharge, cough, rectal temperature (>39.5°C) and the presence of dyspnea as criteria. Herds were visited by the primary investigator between 4 and 8 times during the registration period to check compliance with the recording system. Slaughter data (date of slaughter, hot carcass weight (HCW), carcass quality, color and fat classification) were collected at the slaughter houses. The European SEUROP classification system was used to determine carcass quality (18 classes) and scoring was done visually by trained staff. Meat color was determined by spectrophotometry and the European classification system was used (10 classes) [26].

### Serology

Given the diverse etiology of BRD in calves, paired serology was used to identify the circulating pathogens. In each herd 25 calves were randomly selected at arrival using the official stable lists. Blood samples, taken within the normal management practices in veal production (determination of iron levels) at arrival and 24 weeks later, were used. Serum was collected within 8 hours after sampling and stored at −18°C until analysis. Semi-quantitative indirect ELISA’s were used to detect antibodies (IgG1) against bovine respiratory syncytial virus (BRSV), parainfluenza virus type 3 (PI-3), bovine viral diarrhea virus (BVDV) (NS2-3 native protein), bovine adenovirus type 3 (BAV-3), BHV-1 (Respiratory ELISA kit pentakit, Bio-X Diagnostics, Jemelle, Belgium) and *Mycoplasma bovis* (*M. bovis* ELISA kit, Bio-X). Tests were performed and interpreted according to the manufacturers prescriptions, as outlined in detail elsewhere [27]. Sera from the same calf were tested on the same plate. Antibodies against *Mannheimia haemolytica* (whole cell) were determined at the laboratory of MSD Animal Health (Boxmeer, The Netherlands) with an in-house ELISA [28]. Dilution series of the sera were incubated on plates and bound antibodies were detected after incubation with an anti-bovine serum-peroxidase conjugate [28]. A four-fold titer increase was considered a seroconversion. All methods used in this study were in concordance with the ethical conditions for animal experimentation as mentioned in the Belgian (KB 14 August 1986) and European legislation (Directive 86/609/EEC).

### Data management

Mortality, treatment and slaughter data were entered in a relational data base (Access 2007, Microsoft Inc.) and transferred to SAS version 9.3 (SAS Institute Inc., Cary, NC) for descriptive and statistical analysis. A calf was considered a case of a given disease (respiratory disease, diarrhea, otitis and arthritis), when individually diagnosed and treated by the producer or veterinarian for that indication on at least one day. For respiratory disease, a calf could experience several episodes. Each newly started treatment course more than 14 days after the preceding treatment, was counted as a new episode [17,29]. A relapse case is defined as an animal, which experienced 2 or more BRD episodes. The long acting effect of certain antimicrobial formulations was taken into account by counting one injection as 2 (tilmicosin, amoxicillin, florfenicol, danofloxacin) or 9 (tulathromycin) days of treatment [17,29]. The mortality risk was calculated as the number of death calves divided by the number of calves at risk at calf arrival [17]. The directors of the slaughterhouses were asked which SEUROP score of fat cover, carcass quality and carcass color they judged as insufficient for white veal production. Based on this judgment, binary outcome variables were constructed for these variables. Fat cover was split into carcasses with no fat (European class 1) and normal to extremely fat carcasses (European class 2 to 5). All carcasses graded P + (European class 13) and lower were grouped as insufficient carcass quality and analyzed as such. For meat color two classes were created, representing white meat (European class 1 to 6) and meat with an undesirable red color (European class 7 to 10) for white veal production. Hot carcass weight (HCW) (kg) was measured at the slaughterhouse at 0.1 kg precise. Drug use was determined according to standard daily dose methodology [24]. For modeling purposes, the percentage of the production cycle that calves spent on oral antimicrobial drugs (= antimicrobial drug use (ADU)) was calculated for each individual calf. ADU was calculated as the number of days spent on oral antimicrobials/total number of days on feed (DOF) x 100. DOF was calculated on an individual basis by subtracting the arrival date from the slaughter date.

In order to estimate the economic consequences of the observed disease effects, the average prices per kg HCW from years 2008–2009, available from the veal industry, were used. These were 4.6 €/kg (standard deviation (SD) = 0.5) for BHF, 5.3 €/kg (SD = 1.0) for RHF, 6.4 €/kg (SD = 1.0) for crossbreds and 8.1 €/kg (SD = 0.8) for BB. Meat with an undesirable red color returned 0.1 €/kg, 0.2 €/kg, 0.4 €/kg and 0.2 €/kg less compared to an adequate pale color of the carcass for BHF, RHF, crossbreds and BB, respectively. Carcasses with a European fat grading score of 1 (very skinny) returned 0.1 €/kg, 0.2 €/kg, 0.0 €/kg for BHF, RHF and crossbreds, respectively. In contrast BB carcasses with a fatness degree of 1 returned 0.4 €/kg more. Carcasses with a SEUROP score of 13 or less returned 0.1 €/kg, 0.3 €/kg, 1.7 €/kg and 0.3 €/kg less compared to higher scores, for BHF, RHF, crossbreds and BB, respectively. The total treatment cost per calf (in €) included the costs related to both group and individual treatments. The cost of oral group treatments was calculated for each herd from the official treatment records, which are under supervision of the federal agency responsible for safety of the food chain, using the average price (€/kg product) of each drug from the period 2008–2009, available from the veterinary practices who sold these products. The cost of individual treatment for each calf was calculated by multiplying the number of individual treatment days by the average cost of one individual treatment day. An individual treatment day was defined as each day on which an individual calf received one or more individual treatments. The cost of an individual treatment day was calculated by dividing the cost of all individually administered drugs in a herd by the total number of individual treatment days for that herd. Feed cost, labor, housing costs and governmental supports were not taken into account for cost calculation.

### Statistical analysis

The unit of analysis was the individual calf. To estimate the effect of the studied diseases (BRD, diarrhea, arthritis and otitis) on HCW a linear mixed model with herd as random factor to account for clustering of calves within a herd was used (PROC MIXED). First the continuous outcome variable (HCW) was checked for a normal distribution. Univariable associations between predictor variables (BRD, diarrhea, otitis, arthritis, age at arrival, breed, gender and ADU) were explored by univariable logistic (PROC GLIMMIX) regression for binary outcomes and by ANOVA for continuous variables (PROC MIXED). Next all predictor (studied diseases, breed, age at arrival, gender and ADU) variables were tested univariably for their association with HCW. All predictors with *P* < 0.2 in the univariable model were maintained for the multivariable model, which was built stepwise backward, gradually excluding non-significant variables. Before entering the predictor variables in the multivariable model Pearson and Spearman’s rho correlations were calculated and when correlation was higher than 0.60, only the most significant variable was retained. For the final models, pairwise comparisons for categorical predictors were made, with Bonferroni adjustment for multiple comparisons. All biologically relevant two-way interactions of significant fixed effects were tested. Significance was set at *P* < 0.05 and *P* < 0.10 was considered a trend. When necessary to make the models convert, otitis and arthritis were combined into one variable. Model fit and assumptions were evaluated by (graphically) checking the normal distribution of the residuals.

The effect of the different calf diseases on low fat cover (0/1), red meat color (0/1), and low carcass quality (0/1) was analyzed by multivariable logistic regression. A generalized linear mixed model (PROC GLIMMIX) was used with binomial distribution and logit link function with Wald’s statistics for type 3 contrasts. Herd was added as a random factor to account for clustering. First, the same predictors as for the mixed model, were tested univariably. All predictors with *P* < 0.2 in the univariable model were maintained for the multivariable model, which was built stepwise backward, gradually excluding non-significant variables. For the final models, pairwise comparisons for categorical predictors were made, with Bonferroni adjustment for multiple comparisons. All biologically relevant two-way interactions of significant fixed effects were tested. Significance was set at *P* < 0.05 and *P* < 0.10 was considered a trend. When necessary to make the models convert, otitis and arthritis were combined into one variable. Model fit was evaluated by the Hosmer-Lemeshow goodness-of-fit test for logistic models [30].

To visualize the relationship between the studied disease and mortality over time, first Kaplan-Meier survival curves (PROC LIFETEST) were created, disregarding the fact that there might be a dependence of the herd. In a next step a multivariable Cox proportional hazards model (PROC PHREG) was built, with a frailty term to account for clustering of calves within a herd. DOF (days) was used as the survival time and mortality (0/1) as the censor variable. Right censoring occurred at the date of slaughter. Exposures considered were BRD, diarrhea, arthritis, otitis, age at arrival, breed and gender. First all predictors were tested univariably, and those with *P* < 0.20 were withhold for the multivariable model. This model was built stepwise backwards, gradually excluding none-significant variables. All biologically relevant two-way interactions of significant fixed effects were tested. Significance was set at *P* < 0.05 and *P* < 0.10 was considered a trend. Wald’s test was used to assess parameter estimate significance. Visual inspection of the log-cumulative hazard plots and the Schoenfeld residuals was used to evaluate compliance with the assumptions of proportional hazard models [30]. Because the disease risk varies over time, the assumption of proportional hazards is likely violated. Therefore, a second approach was made adding the diseases as time-varying covariates instead of fixed factors. The same model building strategy as for the model with fixed factors was used.

## Results

### Animals and serology

Mean size of the studied cohorts was 351.9 calves (SD = 121.6; Range (R) = 166–570). Of the 3519 veal calves, 36.8% was BHF, 3.5% RHF, 34.5% BB and 25.1% crossbreds. The majority of the calves was male (91.0%; 3202/3519). The proportion of females was higher in red HF (13.7%; 17/124), BB (9.1%; 111/1215) and crossbreds (16.4%; 145/884) than in black HF (3.4%; 44/1296) (*P* < 0.001). The mean age at arrival was 18 days (SD = 4.8; R = 4–41). Of the calves, 4.1% was of non-Belgian origin and these were exclusively dairy calves. The Belgian calves originated from multiple herds with an average of 1.3 (SD = 0.1; R = 1.2-1.5) delivered calves per herd of origin. The mean time spent in production (DOF) was 192.0 days (SD = 33.5; R = 0–281), and was longer in crossbreds (196.5; SD = 31.9) and BB’s (196.0; SD = 37.6) than in red (180.7; SD = 47.0) or black HF (186.2; SD = 27.2) (*P* < 0.001). *M. bovis*, BVDV and BAV-3 were the most prevalent pathogens in the studied cohorts (Table 1).

**Table 1:** **Seroconversion risk**^**a **^**for respiratory pathogens in 10 white veal cohorts in Belgium**, **2008-2009**

**Pathogen**	**Seroconversion risk (%) (mean ± SD)**	**Range (min.- max.)**	**Herds affected (%)**
Bovine respiratory syncytial virus	8.4 ± 11.4	0-36	70
Parainfluenzavirus type 3	21.2 ± 9.8	12-40	100
Bovine viral diarrhea virus	57.6 ± 27.1	0-84	90
Bovine herpesvirus type 1	3.2 ± 5.9	0-16	30
Bovine adenovirus type 3	50.8 ± 17.1	36-88	100
*Mycoplasma bovis*	79.6 ± 13.7	56-96	100
*Mannheimia haemolytica*	32.4 ± 26.4	0-76	80

^a^Seroconversion risk = number of animals within a cohort that seroconverted/total number of calves present in that cohort x 100; SD = standard deviation.

### Morbidity and mortality

Based on standard daily dose methodology, the average treatment incidence was 407.8 animal daily dosages per 1000 calves at risk [24]. This means that the studied calves received enough oral antimicrobials to treat them for 41,4% of the production cycle length. However, in reality, due to the frequent combination of multiple antimicrobials into one oral treatment, calves received antimicrobials in the milk for on average 25.2% (SD = 10.0; Range (R) = 10.3-45.0) of the time (=ADU). Further details on drug use are available elsewhere [24]. In addition to the metaphylactic group treatments, 22.7% (798/3519) of the calves was individually treated for one or more diseases. Of the calves, 14.8% (522/3519) were individually diagnosed and treated for BRD, 5.3% (186/3519) for diarrhea, 1.5% (52/3519) for arthritis and 1.6% (56/3519) for otitis. The average BRD incidence at the cohort level was 17.2%, ranging from 8.2-33.9%. Of the calves, 1.7% (59/3519) experienced 2 BRD episodes and 0.3% (12/3519) 3 or more episodes. The average day of first treatment for BRD, diarrhea, arthritis and otitis was 41.2 (SD = 39; Median (M) = 27.5; R = 1–287), 12.2 (SD = 27.1; M = 6; R = 1–273), 56.5 (SD = 174; Median = 43; R = 2–174) and 63.0 (SD = 43.2; M = 55; R = 3–231) days after arrival, respectively. Older calves at arrival had less risk to develop diarrhea (odds ratio (OR) = 0.95 per day increase in age; 95% confidence interval (CI) = 0.91-0.98; *P* < 0.01), whereas neither age at arrival, gender or breed influenced the occurrence of BRD, otitis and arthritis. Calves with diarrhea had higher risks for BRD (OR = 2.8; CI = 2.0-3.9; *P* < 0.001) and BRD was associated with increased risks to develop arthritis (OR = 2.2 ; CI = 1.2-4.2; *P* < 0.05) and otitis (OR = 2.4; CI = 1.3-4.2; *P* < 0.01).

**Figure 1:**
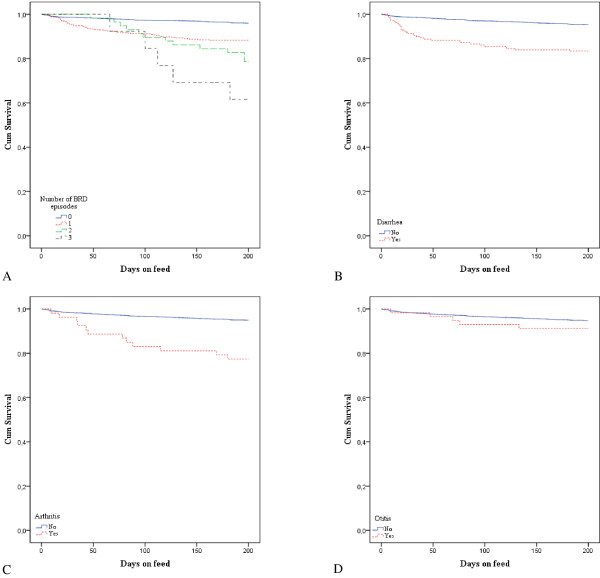
Survival distribution functions for mortality in 10 commercial veal herds in Belgium, including calves (n = 3519) individually diagnosed and treated for bovine respiratory disease (A), diarrhea (B), arthritis (C) and otitis (D).

The number of individual treatment days was on average 5.6 days (SD = 5.9) per treated calf, ranging from 1 to 46. The mean cost of individual treatment in these individually treated calves was €50.9 (SD = 51.1; M = 33.8; R = 8.5-270.4). The mean cost of the oral group treatments was €7.5 (SD = 3.8; R = 2.8-13.5). Descriptives of all outcome variables are given by disease in Table 2. Overall, 5.7% (199/3519) of the calves died before the end of the production cycle, of which 27.1% (54/199) was classified as pneumonia, 7.5% (15/199) as enteritis and 3.5% (7/199) as arthritis. Other important causes of death were acute ruminal disorders (11.0%), enterotoxaemia (10.0%), idiopathic peritonitis (7.0%), death at arrival (5.0%), omphalitis (2.5%) and perforating abomasal ulceration (2.5%) (10.0% of the calves was not autopsied). No calves died from otitis only. Of the calves, that died from pneumonia, 66.7% (36/54) had been individually treated for BRD. Fatal cases of enteritis and arthritis were individually treated for the respective disease in 40.0% (6/15) and 71.4% (5/7) of the cases, respectively. In Figure 1, the survival curves for the studied diseases are shown. In the proportional hazards model with the diseases added as fixed factors, all diseases, except for otitis, were associated with a higher mortality risk (*P* < 0.001) (Table 3). The mortality risk markedly increased with increasing number of BRD episodes (*P* < 0.001). When adding the studied diseases as time-varying covariates two important changes were noted (Table 4): otitis became significantly associated with mortality and the hazard ratio for diarrhea markedly increased (from 2.8 to 11). In both models female calves were less likely to die (*P* < 0.03) and red HF showed a higher mortality risk compared to all other breeds (*P* < 0.03) (Tables 3 and 4).

**Table 2:** **Mortality and carcass traits by disease history in 3519 white veal calves**, **housed in 10 Belgian herds**, **2008-2009**

**Disease**	**Level**	**Calves (n)**	**Mortality**	**HCW (kg) Mean ± SD**	**Low fat cover**^**a**^	**Red meat color**^**b**^	**Low carcass quality**^**c**^
			**% (number)**	**(min.-max.)**	**% (number)**	**% (number)**	**% (number)**
Number of BRD episodes	None	2997	4.3% (128/2997)	172.6 ± 33.0 (61.0-277.3)	6.1% (160/2629)	14.7% (386/2629)	10.3% (272/2637)
	1	451	12.0% (54/451)	163.3 ± 30.6 (79.8-246.2)	10.6% (39/367)	15.0% (55/367)	7.8% (29/370)
	2	59	22.0% (13/59)	142.5 ± 38.2 (81.0-251.0)	10.8% (4/37)	27.0% (10/37)	27.0% (10/37)
	≥ 3	12	33.3% (4/12)	137.6 ± 36.5 (93.8-189.0)	28.6% (2/5)	14.3% (1/7)	42.9% (3/7)
Diarrhea	No	3333	5.1% (169/3333)	171.3 ± 33.3 (61.0-277.3)	6.7% (196/2907)	15.1% (440/2907)	10.3% (300/2917)
	Yes	186	16.1% (30/186)	164.5 ± 30.1 (87.7-244.9)	6.8% (9/133)	9.0% (12/133)	10.4% (14/134)
Otitis	No	3463	5.6% (194/3463)	171.4 ± 33.1 (61.0-277.3)	6.9% (205/2992)	15.0% (448/2992)	10.3% (308/3003)
	Yes	56	8.9% (5/56)	146.8 ± 27.5 (98.9-246.2)	0.0% (0/48)	8.3% (4/48)	12.5% (6/48)
Arthritis	No	3467	5.4% (187/3466)	171.1 ± 33.1 (61.0-277.3)	6.7% (202/3014)	15.0% (452/3014)	10.3% (312/3025)
	Yes	52	23.1% (12/52)	167.2 ± 39.2 (65.9-226.7)	11.5% (3/26)	0.0% (0/26)	7.7% (2/26)

HCW = hot carcass weight; SD = standard deviation; BRD = bovine respiratory disease; ^a^European SEUROP class 1 regarded as insufficient fat cover; ^b^European SEUROP class 7 to 10 regarded as undesirable red meat color; ^c^European SEUROP class 13 (P^+^) and lower.

**Table 3:** **Proportional hazard model for mortality in 3519 white veal calves**, **housed in 10 commercial veal herds in Belgium with the studied diseases added as fixed factors**

**Variable**	**Level**	**Calves (n)**	**ß**	**SD**	**HR**	**HR (95% CI)**	***P*-value**
Calf gender	Male (ref)	3202	0	-			
	Female	317	−0.8	0.4	0.4	0.2-0.9	0.04
Breed							0.04
	Black HF (ref.)	1296	0	-			
	Red HF	124	0.7	0.3	2.1	1.1-5.5	0.03
	Crossbreds	884	−0.2	0.3	0.8	0.5-1.4	0.44
	BB	1215	−0.3	0.3	0.7	0.4-1.4	0.33
Number of BRD episodes							<0.001
	None (ref.)	2997	0	-			
	1	451	1.0	0.2	2.6	1.9-3.6	<0.001
	2	59	1.4	0.3	4.1	2.2-7.8	<0.001
	≥ 3	12	1.9	0.5	6.5	2.5-16.5	<0.001
Diarrhea	No (ref.)	3333	0	-			
	Yes	186	1.0	0.2	2.8	1.9-4.2	<0.001
Arthritis	No (ref.)	3467	0	-			
	Yes	52	1.1	0.3	2.9	1.6-5.3	<0.001
Herd effect ^a^			0.3	0.2			<0.001

SD = standard deviation; HR = hazard ratio; BRD = bovine respiratory disease; HF = Holstein Friesian; BB = Belgian Blue; ref. = referent category; ^a^frailty term.

**Table 4:** **Proportional hazard model for mortality in 3519 white veal calves**, **housed in 10 commercial veal herds in Belgium with the studied diseases added as time-varying covariates**

**Variable**	**Level**	**Calves (n)**	**ß**	**SD**	**HR**	**HR (95% CI)**	***P*-value**
Calf gender	Male (ref)	3202	0	-			
	Female	317	−0.8	0.4	0.5	0.2-0.9	0.04
Breed							0.05
	Black HF (ref.)	1296	0	-			
	Red HF	124	0.8	0.3	2.1	1.1-4.2	0.03
	Crossbreds	884	−0.2	0.3	0.8	0.5-1.4	0.50
	BB	1215	−0.3	0.3	0.8	0.4-1.4	0.38
BRD	No (ref.)	2997	0	-			
	Yes	522	1.7	0.3	5.5	3.0-10.2	<0.001
Diarrhea	No (ref.)	3333	0	-			
	Yes	186	2.4	0.4	11.0	5.4-22.6	<0.001
Arthritis	No (ref.)	3467	0	-			
	Yes	52	1.7	0.9	3.9	1.6-5.3	0.05
Otitis	No (ref.)	3463	0	-			
	Yes	56	1.9	0.9	7.0	1.3-38.5	0.03
Herd effect ^a^			0.3	0.2			<0.001

SD = standard deviation; HR = hazard ratio; BRD = bovine respiratory disease; HF = Holstein Friesian; BB = Belgian Blue; ref. = referent category; ^a^frailty term.

### Carcass traits

HCW was available for 3210 calves. The remaining calves died during production (n = 199) or were live exported (n = 110). The mean HCW was 171.0 kg (SD = 33.2) ranging from 61.0 to 277.3 kg (red HF = 148.9 (SD = 20.1); Black HF = 152.0 (SD = 24.1); Crossbreds = 176.1 (SD = 27.7); BB = 194.1 (SD = 32.1)). Of the variation in HCW, 48% was situated at herd level and 52% at the individual calf level. In a first model all diseases were added separately, not taking the number of BRD episodes into account (Table 5). Breed, gender and ADU were significantly associated with HCW. With every increase of ADU by 1%, HCW reduced by 1.5 kg on average. BRD and diarrhea (*P* < 0.001) were associated with marked weight loss, respectively 9.7 kg and 9.2 kg. In this model the interactions between arthritis and BRD (*P* < 0.01) and arthritis and otitis (*P* = 0.01) were significant. HCW of arthritis cases only differed significantly from apparently healthy calves when also treated for BRD. The second interaction was situated in the fact that only arthritis cases, which did not have otitis, differed significantly from the control group. Otitis as such was not associated with a reduced slaughter weight. The three way interaction between BRD, arthritis and otitis did not converge. The final model as shown in Table 5 explained 6.0% and 15.4% of the variation in HCW at calf and herd level, respectively. A second model was made in order to assess the effect of the number of BRD episodes on HCW, using a combining variable for otitis and arthritis to make the model converge. HCW decreased severely with increasing number of BRD episodes, namely with on average 8.2 kg, 22.4 kg and 41.6 kg in calves, which experienced 1, 2 or ≥3 BRD episodes, respectively (*P* < 0.001) (Table 6). This model showed a slightly better fit and explained 6.0% and 9.0% of the variation at calf and herd level, respectively.

**Table 5:** **Final linear mixed model with pairwise comparisons for the effect of the most frequent calf diseases on hot carcass weight (HCW) (kg) of 3210 white veal calves**, **housed in 10 herds in Belgium**, **2008-2009**

**Variable**	**Level**	**Reference**	**ß**	**SD**	***P*-value**
Breed					< 0.001
	Red HF	Black HF	−4.5	2.4	0.36
	Crossbreds	Black HF	3.1	1.7	0.42
	BB	Black HF	10.4	2.1	<0.001
	Crossbreds	Red HF	7.6	2.5	0.02
	BB	Red HF	15.0	2.8	<0.001
	Crossbreds	BB	7.3	1.5	<0.001
ADU (%)			−1.5	0.4	< 0.001
Calf gender	Female	Male	−10.2	1.6	<0.001
Diarrhea	Yes	No	−9.2	2.0	<0.001
BRD	Yes	No	−39.8	10.4	<0.001
Arthritis	Yes	No	7.7	16.9	0.65
Otitis	Yes	No	39.8	19.0	<0.01
BRD x ART					<0.01
	No BRD/ART	No BRD/No ART	14.3	10.6	1.0
	BRD/No ART	No BRD/No ART	−9.7	1.3	<0.001
	BRD/ART	No BRD/No ART	−25.6	9.7	0.05
	BRD/No ART	No BRD/ART	−24.0	10.6	0.15
	BRD/ART	No BRD/ART	−39.8	10.4	<0.001
	BRD/ART	BRD/No ART	−15.9	9.8	0.62
OTI x ART					0.01
	No OTI/ ART	No OTI/No ART	−24.4	5.3	<0.001
	OTI/No ART	No OTI/No ART	−7.4	3.5	0.21
	OTI/ART	No OTI/No ART	15.4	17.3	1.0
	OTI/No ART	No OTI/ART	17.1	6.3	0.04
	OTI/ART	No OTI/ART	39.8	19.0	0.22
	OTI/ART	OTI/No ART	22.8	17.6	1.0

SD = standard deviation; BB = Belgian Blue; HF = Holstein Friesian; ADU = antimicrobial drug use expressed as the number of days on oral antimicrobials/total number of days on feed (%); BRD = bovine respiratory disease; ART = arthritis; OTI = otitis; x = interaction.

**Table 6:** **Final linear mixed model with pairwise comparisons for the effect of the number respiratory disease episodes and other diseases on hot carcass weight (HCW) (kg) in 3210 white veal calves**, **housed in 10 herds in Belgium**, **2008-2009**

**Variable**	**Level**	**Reference**	**ß**	**SD**	***P*-value**
Breed					<0.001
	Red HF	Black HF	−4.4	2.4	0.4
	Crossbreds	Black HF	3.0	1.8	0.48
	BB	Black HF	10.4	2.1	< 0.001
	Crossbreds	Red HF	7.4	2.5	0.02
	BB	Red HF	14.8	3.0	< 0.001
	Crossbreds	BB	7.4	1.5	<0.001
ADU (%)			−1.6	0.4	<0.001
Calf gender	Female	Male	−10.2	1.6	<0.001
Diarrhea	Yes	No	−9.2	2.0	< 0.001
ART and/or OTI	Yes	No	−9.1	2.6	< 0.001
BRD					< 0.001
	1 episode	No BRD	−8.2	1.3	<0.001
	2 episodes	No BRD	−22.4	3.8	<0.001
	≥3 episodes	No BRD	−41.6	8.3	< 0.001
	2 episodes	1 episode	−14.2	3.9	< 0.001
	≥3 episodes	1 episode	−33.4	8.4	<0.001
	≥3 episodes	2 episodes	−19.3	9.1	0.20

SD = standard deviation; BB = Belgian Blue; HF = Holstein Friesian; ADU = antimicrobial drug use expressed as the number of days on oral antimicrobials/total number of days on feed (%); BRD = bovine respiratory disease; ART and/or OTI = combining variable for calves treated for arthritis (ART) and/or otitis (OTI).

Meat color was available for 3040 calves. The odds for an undesirable red meat color trended to be larger in calves, which relapsed for BRD (≥2 episodes) (OR = 2.5; CI = 1.0-6.4; *P* = 0.06) and increased with increasing age (OR = 1.04 per day increase in age; CI = 1.01-1.06; *P* < 0.01). In contrast, the combining variable of arthritis and otitis was associated with a lower risk (OR = 0.25; CI = 0.09-0.71; *P* < 0.01). With an increasing percentage of the production time spent on oral antimicrobials (=ADU), the odds for too red meat decreased (OR = 0.86 per percentage increase in ADU; CI = 0.76-0.98; *P* < 0.05). Also, female calves trended to have a higher odds for red meat color (OR = 1.4; CI = 0.9-2.1; *P* = 0.10). Fat cover (available for 3040 calves) was only affected by BRD, with calves treated once (OR = 2.5; CI = 1.5-4.2; *P* < 0.001) and relapse cases (OR = 3.8; CI = 1.0-15.1; *P* = 0.06) being more likely to have carcasses with too low fat cover. Also, with an increasing percentage of the production time spent on oral antimicrobials (=ADU), the odds for insufficient fat cover decreased (OR = 0.88 per percentage increase in ADU; CI = 0.78-0.99; *P* < 0.05). Carcass quality was available for 3051 calves. Calves which experienced diarrhea (OR = 2.5; CI = 1.2-5.4; *P* < 0.05) or relapsed for BRD (two episodes vs. none (OR = 10.9; CI = 3.1-38.5; *P* < 0.001) and three or more episodes vs. none (OR = 50.0; CI = 3.6-333.3; *P* < 0.001)) had higher odds for low carcass quality (SEUROP score P + or lower). Also, BB calves had lower odds (OR = 0.07; CI = 0.01-0.42; *P* < 0.001) for low carcass quality compared to red HF.

### Economic considerations

From the regression models and based on the average prices from 2008–2009, as provided in this article, estimations of the economic consequences of the studied diseases could be made. For example, in calves which experienced 1 BRD episode the average loss in HCW of 8 kg signifies a financial loss of €36.8, €42.4, €51.2 and €64.8 in BHF, RHF, crossbreds or BB calves, respectively. In a BHF veal calf, which experienced three BRD episodes, on average 41.6 kg of carcass weight is lost, representing €187.2. This calf also has higher odds for low carcass quality and too red meat color. When taking into account that this calf weighs on average 110.4 kg (41.6 kg less than the average BHF calf), an additional loss of €11.4 for low carcass quality and also €11.4 due to a too red meat color can be expected. This brings the total loss to €210, to which the costs of group and individual antimicrobial treatment still need to be added. For comparison, in BB calves, which have a much higher meat price due to the excellent carcass characteristics, the same calf would signify a financial loss as high as €622.3.

## Discussion

The present study aimed at determining the long term effect of different calf diseases on important economic parameters in white veal production. Unfortunately, nutrition, which is known to explain the greatest proportion of variation in HCW, could not be included in the models [31,32]. The reasons were that the different integrations were not willing to provide the composition of the diet and that the commercial veal stables were not adapted to measure feed intake individually. Nevertheless, the present study shows that the studied diseases, breed and gender explain a substantial proportion of the variation in HCW at calf and herd level.

As in most epidemiological studies on BRD, producer based diagnosis was used, because this approach is closest to realistic on farm procedures and therefore better interpretable by the industry itself [3,4,9,10,33-35]. For diarrhea, arthritis and otitis, straightforward case definitions based on obvious symptoms were provided to the producers, and reporting bias is believed to be limited for these diseases. For BRD the situation is more complicated, as it is well known that based on clinical examination both farmers as veterinarians tend to detect only a proportion of the cases and rather late in the disease process [36,37]. Especially the initial symptoms of an infection with *M. bovis*, the dominant pathogen in the veal industry, are very subtle, even when extensive pneumonia is already present, and hard to evaluate in individual housing [38]. An additional issue in the veal industry is the frequent use of pro- and metaphylactic antimicrobial group treatment, which interferes with the recognition of individual disease [39]. Also, in the present study, one third of the calves, which showed extensive pneumonia at necropsy, has not been individually diagnosed previously. For these reasons and despite that the BRD incidence was in line with what has been reported for feedlots (17.0% on average, ranging from 4.6-43.8%) and even higher than reported in veal calves in the Netherlands, Italy and France (<7%), the BRD incidence is likely underestimated in the present study [5,39]. Therefore, the results and associations documented in this study should be interpreted as representing animals with obvious clinical symptoms, with onset of BRD before the installment of the metaphylactic group treatment or calves non-responding to oral group treatment. The overall economic loss due to BRD is likely greater than demonstrated in the present study.

When looking at the short term consequences of BRD, significant weight loss in the three weeks following disease has been demonstrated both in feedlot cattle (−0.370 kg/day) and veal calves (−0.070 kg/day to −0.280 kg/day depending on the installed treatment) [16,23]. When analyzed over the complete production cycle the loss in average daily gain (ADG) is less pronounced (e.g. -0.070 kg/day in feedlots), signifying that after the period of clinical BRD shortly after arrival, subsequent compensatory weight gain occurred in treated animals, possibly because BRD cases show a higher eating frequency after disease [23,40]. In South African feedlots this weight loss was shown to be fully compensated, but in most other feedlot studies a significant reduction in HCW or ADG was still present at slaughter [6,13,23]. Also in the present study a single BRD episode reduced HCW by 8.2 kg on average, which is similar to feedlot cattle [6,23]. However, the relative loss in HCW is higher in white veal calves compared to feedlot cattle (4.9% vs. 2.3%) and has a greater economic significance, because of the higher prices for veal meat [6]. This BRD associated weight loss has been attributed to reduced feed intake due to anorexia and depression and to the increased protein and caloric cost of a febrile response and (chronic) inflammation [40-44].

As in feedlots and dairy calves, also in veal calves the reduction in HCW became more pronounced with an increasing number of BRD episodes [6,8,10,23,45]. Chronic BRD also had a significant negative effect on carcass quality and fat cover, as documented for feedlots [6,9]. In feedlots chronic unresponsive pneumonia has been associated with *Mycoplasma bovis* and bovine viral diarrhea virus [46,47]. Both pathogens were also highly prevalent in the studied herds as documented previously in white veal calves [27,48]. In addition to pneumonia, *M. bovis* causes arthritis and otitis media (*M. bovis* associated disease (MbAD)) [46]. The effects of arthritis on performance have not been specifically reported, despite the high incidence of MbAD in feedlots. In the present study calves with concurrent arthritis and BRD showed extensive weight loss similar to chronic BRD cases, whereas calves with only arthritis did not have a significant lower HCW. Most likely in the latter calves the arthritis was of traumatic origin and healed after treatment, whereas it was associated with chronic *M. bovis* infection in the calves with concurrent BRD. In calves with chronic arthritis feed uptake is likely further reduced, since the painful joints make them reluctant to move to the drinking trough to eat. Otitis media was not associated with decreased growth, as was the case in a dairy heifer raising facility with high incidence of *M. bovis*[12]. Whether this truly means that otitis does not affect performance, or whether the individual antimicrobial treatment has been installed quickly enough by the producers due to the obvious clinical symptoms, hereby alleviating the negative consequences, remains to be determined. When adding otitis as a fixed variable to the survival model, no association with mortality could be demonstrated, as was the case in a previous study on dairy heifers with otitis [12]. In contrast, when working with otitis as a time-varying covariate, a significant effect of otitis on mortality was found. This is related to the fact that an animal that gets otitis has a higher hazard of dying within the 7 days after the infection than in the rest of the at risk period. However, given the relatively low number of events for otitis and arthritis, these findings should be interpreted carefully. Mortality, especially at older age, greatly determines the economic revenues of a group of veal calves. The present study shows that all studied diseases have an important association with this mortality risk. Given that *M. bovis* is associated with BRD, arthritis and otitis, preventive and curative management of this pathogen should have priority. Also, the mortality risk of both chronic BRD and arthritis cases was increased in such a way, that one should question whether treatment of a 3rd BRD episode or arthritis is still economically and ethically justifiable.

In contrast to BRD, few studies have addressed the effects of neonatal calf diarrhea on survival and carcass traits. Diarrhea increased the mortality risk in white veal calves as has been observed in conventional dairy calves in the first 180 days of life [49]. The present study also showed that developing diarrhea, which occurs predominantly in the first 3 weeks after arrival, has a markedly stronger association with mortality than BRD, arthritis or otitis in veal calves. Therefore, management of this disease deserves full attention in veal herds. Further work to clarify which diarrhea pathogens are exactly associated with the highest mortality risk and to identify risk factors for diarrhea is needed in order to be able to install effective control measures. Previously, in veal calves, diarrhea has been associated with significant weight loss in the clinical period [14]. Additionally, the present study shows a significant long term effect of diarrhea on HCW value in veal calves, similar to effects observed in large scale dairy calf rearing (−0.051 kg/day) in the same age period [50]. Whereas in one study in small scale dairy calf rearing full compensation of neonatal diarrhea associated weight loss at the age of 3 months was reported, the present and a previous study in large scale dairy calf rearing demonstrated that weight loss due to neonatal diarrhea is not fully compensated at the age of 6 months [10,50]. It is important to notice that diarrhea also influenced carcass grading, causing additional economic loss. Despite the fact that calves, which had developed diarrhea, were predisposed for BRD, as was seen in other studies, the interaction between BRD and diarrhea was not significant in any model, suggesting that both diseases independently significantly affected HCW [51,52].

Meat color is an important marketing parameter, which greatly determines carcass value in white veal calves [1]. The white color of veal meat is obtained by reducing iron uptake, resulting in lower hemoglobin (Hb) and myoglobin levels. However, meat color is affected by much more factors, since Hb only accounted for 29% of the variation in visual color score [53]. In the present study, a trend was shown that chronic BRD results in an increased probability of undesirable red meat at slaughter. A possible explanation, next to the fact that chronic stress causes dark, firm and dry meat, might be that chronic BRD cases, which often suffer from ruminal drinking as well, are more frequently switched to an alternative, more iron rich, concentrate diet [54]. Other factors associated with red meat were older age at arrival and female gender. Both effects are not straightforward to explain, but possibly they are related to age and gender differences in iron metabolism as demonstrated in humans and rats [55,56]. In the present study we were obliged to use a combining variable for otitis and arthritis to make the model for meat color convert. To fully understand the influence of BRD, otitis and arthritis on meat color, a dataset large enough to test the interaction between these diseases is necessary and present results on arthritis and otitis should be interpreted with care concerning meat color.

One of the most surprising findings in the present study was that an increase in the percentage of the production cycle calves spent on oral antimicrobials was associated with a decrease in HCW. The opposite was to be suspected, since the growth promoting effect of oral antimicrobials is well documented [57-59]. Since 2006, antimicrobial growth promoters are forbidden in Europe (EC 1831/2003), but the trend is that they have at least partly been replaced by an increased use of therapeutic antimicrobials [60,61]. The most likely explanation for the observed negative association between HCW and antimicrobial use, is that more antimicrobial group treatments were used in those herds, which experienced more health problems. This would mean that the systematic use of oral antimicrobial group treatments was unable to completely alleviate the negative consequences of disease on carcass traits in veal calves. In dairy calves, the use of oral antimicrobial group treatments in the milk has been associated with 31% more days with diarrhea in the first 28 days of life, but not with a difference in ADG [62]. To what extent oral antimicrobial use in veal calves disturbs the intestinal flora, causing antibiotic-associated diarrhea and possibly negative effects on performance is currently unknown. In human medicine, antimicrobial use at young age in infants from mothers with a normal body weight has been associated with an increased risk of overweight at the age of 7 [63]. Possibly, the observed positive association between ADU and fat cover in veal calves is due to similar mechanisms. The effect of in feed antimicrobials on fat cover and carcass color in veal calves needs to be further clarified by using more precise and objective measuring techniques (e.g. measuring fat thickness at slaughter or by ultrasound [64,65]) than the commonly used SEUROP classification system as was the case in the present study.

At present, the high levels of antimicrobial use (especially oral antimicrobial group treatments) and resistance in the veal industry are of great public concern [24,66-70]. The present study shows that despite numerous antimicrobial group treatments for BRD and diarrhea, significant production loss still occurred in veal calves, which required individual treatment. Because individual treatment was used as the case definition, this signifies that the applied individual treatment protocols were unable to completely alleviate the consequences of disease. One reason might be the enormous infection pressure, inherent to the production system, which is unable to be completely overcome by antimicrobial treatment alone. Additional reasons might be the timing of metaphylaxis, the use of antimicrobials for which pathogenic bacteria are resistant, underdosing or insufficiently long individual treatment courses resulting in relapse or persistent subclinical pneumonia [17,24,27]. Field studies, evaluating different preventive and therapeutic protocols for their ability to reduce antimicrobial use while maintaining or even improving current production results, are necessary to direct the veal industry towards the most sustainable production strategy.

## Conclusions

Even under the high level of antimicrobial coverage in contemporary veal production, BRD, diarrhea, arthritis and otitis increase the mortality risk and all except otitis have detrimental effects on carcass traits in white veal calves, leading to substantial economic loss. Losses were more pronounced in cases of chronic pneumonia with or without arthritis. Controlling calf health by effective preventive and therapeutic strategies and in particular the prevention of chronic BRD is key for the profitability of veal operations.

## Competing interests

*Mannheimia haemolytica* ELISA’s were financed by MSD Animal Health. The authors declare that they have no competing interests.

## Authors’ contributions

Conception and design of the study: BP and PD; Farm visits and follow up: BP, KDB; Data management and statistical analysis: BP, JD, MH, LD; Drafting and critically revising the manuscript: BP, JD, PD. All authors read and approved the final manuscript.
